# Integrating New Technologies in Lipidology: A Comprehensive Review

**DOI:** 10.3390/jcm14144984

**Published:** 2025-07-14

**Authors:** Carlos Escobar-Cervantes, Jesús Saldaña-García, Ana Torremocha-López, Cristina Contreras-Lorenzo, Alejandro Lara-García, Lucía Canales-Muñoz, Ricardo Martínez-González, Joaquín Vila-García, Maciej Banach

**Affiliations:** 1 Cardiology Department, La Paz University Hospital, Paseo de la Castellana, 28046 Madrid, Spain; ana.torremocha@salud.madrid.org (A.T.-L.); ccontrerasl@salud.madrid.org (C.C.-L.); alejandro.lara@salud.madrid.org (A.L.-G.); lcanalesm@salud.madrid.org (L.C.-M.); rmartinezgonzalez@salud.madrid.org (R.M.-G.); joaquin.vila.garcia.24@gmail.com (J.V.-G.); 2 Facultad de Medicina, Universidad de Málaga, 29071 Málaga, Spain; 3 Cicarrone Center for the Prevention of Cardiovascular Disease, Johns Hopkins University School of Medicine, Baltimore, MD 21287, USA; maciej.banach@umed.lodz.pl; 4 Department of Cardiology and Adult Congenital Heart Diseases, Polish Mother’s Memorial Hospital Research Institute, 29-071 Lodz, Poland

**Keywords:** lipidomics, dyslipidemia, cardiovascular disease, telemedicine, artificial intelligence, precision medicine

## Abstract

Cardiovascular disease remains the world’s leading cause of death, and even when patients reach guideline low-density lipoprotein cholesterol targets, a substantial “residual risk” persists, underscoring the need for more nuanced assessment and intervention. At the same time, rapid advances in high-resolution lipidomics, connected point-of-care diagnostics, and RNA- or gene-based lipid-modifying therapies are transforming what clinicians can measure, monitor, and treat. Integrating multimodal data through machine learning algorithms capable of handling high-dimensional datasets has the potential to improve cardiovascular risk prediction and re-stratification compared to traditional models. This narrative review therefore sets out to (i) trace how these emerging technologies expand our understanding of dyslipidemia beyond the traditional lipid panel, (ii) examine their potential to enable earlier, more personalized and durable cardiovascular risk reduction, and (iii) highlight the scientific, regulatory and ethical hurdles that must be cleared before such innovations can deliver widespread, equitable benefit.

## 1. Introduction

Cardiovascular disease (CVD) has been, and continues to be, the leading cause of death worldwide. In 2022, it accounted for more than nine million deaths and was the primary contributor to years of life lost due to disability [1]. Although age-adjusted mortality rates have declined over recent decades, population aging and demographic growth are driving a steady increase in the overall burden of CVD. Between 2025 and 2050, cardiovascular deaths are projected to rise by 73%, reaching 35 million annually, while the prevalence of CVD is expected to nearly double [2] (Table 1). Moreover, a disproportionate impact is anticipated across regions, sexes, and socioeconomic levels, along with a sharp rise in healthcare costs, which could exceed $1.3 trillion annually in countries like the United States by 2050 [2].

Among modifiable risk factors, dyslipidemia ranks as the second most relevant metabolic contributor to the prevention of CVD, following hypertension [2]. The evidence is clear: elevated levels of low-density lipoprotein cholesterol (LDLc) cause atherosclerosis and increase the risk of cardiovascular events, making lipid control a major therapeutic priority [3]. For decades, the management of dyslipidemia has been largely “LDL-centric”; however, in recent years, this approach has evolved rapidly with the emergence of new biomarkers, advanced therapies, and digital tools that enable more precise and personalized lipid management.

**Figure 1:**
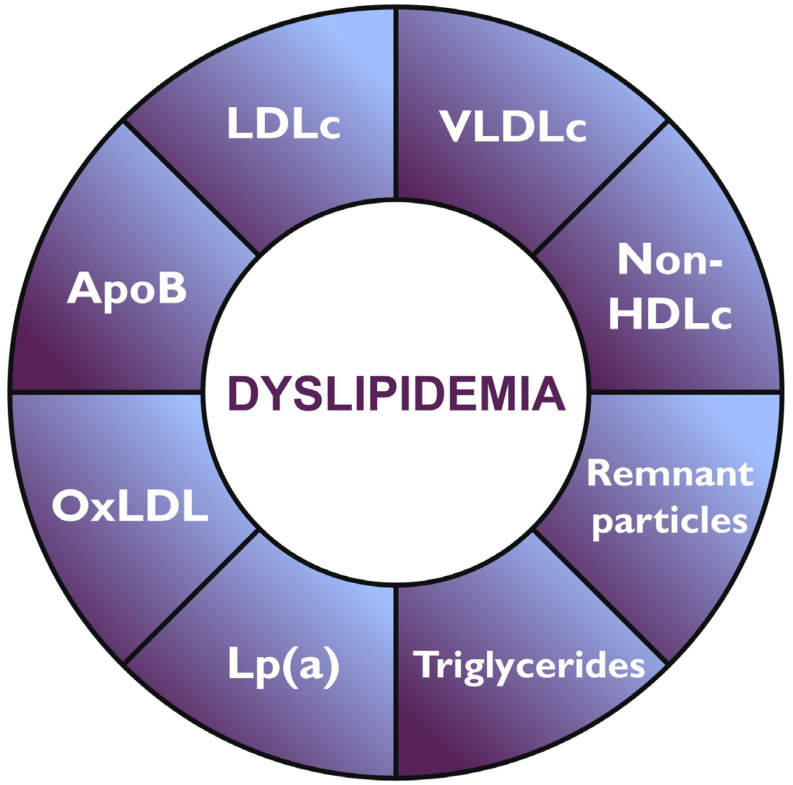
Key lipid-related biomarkers involved in dyslipidaemia. LDL: Low-density lipoprotein; VLDL: Very-low-density lipoprotein; HDL: High-density lipoprotein; Lp(a): Lipoprotein (a); oxLDL: Oxidized LDL; ApoB: Apolipoprotein B.

A significant cardiovascular risk persists despite achieving optimal LDLc targets, a phenomenon known as residual risk [4]. This has prompted the investigation of new biomarkers (Figure 1) that enable more accurate risk assessment. Apolipoprotein B (ApoB), which reflects the total number of atherogenic particles, and non-high-density lipoprotein (non-HDLc) cholesterol have proven to be more robust predictors in certain contexts. In addition, other markers such as lipoprotein(a) [Lp(a)] and remnant particles, triglyceride-rich metabolic byproducts, have been independently associated with residual cardiovascular risk [4,5]. Moreover, oxidation of lipid particles, particularly LDLc, enhances their atherogenic potential. The measurement of oxidized LDLc has been linked to greater atherosclerotic progression and a higher incidence of cardiovascular events, even among patients treated with statins [4].

The development of new diagnostic tools has significantly expanded our ability to characterize the lipid profile and its clinical implications. Computed tomography (CT) enables quantification of coronary artery calcium as well as epicardial fat, an emerging marker of atherosclerosis associated with local inflammation [6]. Techniques such as magnetic resonance spectroscopy (MRS) and mass spectrometry allow for highly precise analysis of the number, size, and composition of lipoprotein particles [7]. Lipidomics, based on these platforms, has identified multiple lipid species associated with the progression of atherosclerosis and cardiovascular events. This data can be integrated with other multi-omic panels through machine learning algorithms capable of handling high-dimensional datasets. This strategy has been shown to improve cardiovascular risk prediction and re-stratification with greater accuracy and personalization compared to traditional clinical models [8]. The digital revolution and the rise of personalized medicine have also driven the adoption of wearable devices, mobile applications, and artificial intelligence (AI) algorithms that support continuous monitoring and early risk stratification, allowing for the identification of individualized risk profiles at earlier stages [9].

Technological advances have also had a major impact on the therapeutic landscape, with the emergence of new molecules such as PCSK9 inhibitors and RNA-based therapies, which have expanded treatment options and enabled better lipid control with more convenient dosing regimens [10]. Additionally, technologies such as gene editing (CRISPR-Cas9) and therapeutic nanotechnology are emerging as disruptive strategies for the treatment of hereditary dyslipidemias and forms resistant to conventional therapies [11].

This document presents a critical review of the most relevant advances in the diagnosis, monitoring, and treatment of dyslipidemia, with a particular focus on the integration of new technologies. The aim is to provide a comprehensive overview of the current state and future perspectives at the intersection of lipidology and technology, within the framework of precision medicine.

## 2. Methods

A narrative literature review was conducted to explore the most recent and relevant advances in technologies applied to lipidology. The review included original research articles, clinical trials, meta-analyses, and systematic reviews published until May 2025. A structured search was performed using PubMed/MEDLINE, Embase Scopus, SciELO and Web of Science databases with combinations of the following keywords: “cholesterol”, “low-density lipoprotein”, “high-density lipoprotein”, “lipoprotein a”, “lipidomics”, “lipoproteins”, “lipid biomarkers”, “wearable devices”, “biosensors”, “telemedicine”, “RNA therapies”, “PCSK9 inhibitors”, “CRISPR-Cas9”, “nanotechnology”, “artificial intelligence”, “deep learning”, “neural network”, and “machine learning”. Preprints were considered only if they presented significant or pioneering findings.

Eligibility was determined based on relevance to the integration of novel technologies in lipid management, including diagnostic tools, treatment modalities, and digital health applications.

The selected studies were critically appraised and grouped into thematic domains reflecting the structure of this review: advanced lipid profiling, diagnostic devices, remote monitoring technologies, emerging therapies, artificial intelligence applications, and clinical decision support systems. Reference lists of included articles and recent reviews were manually screened to identify additional pertinent studies.

## 3. Advances in Lipid Profiling

Atherosclerosis is the underlying mechanism of vascular disease development, and its relationship with lipoproteins is well recognized. Traditionally, cardiovascular risk assessment and treatment decisions have relied on standard plasma lipid measurements such as total cholesterol (TC), LDLc, HDLc, and triglycerides. However, achieving therapeutic targets has not been sufficient for avoiding recurrent vascular events. This is probably related to the inability of these conventional parameters to accurately estimate the individual atherogenic risk, particularly in patients with discordant lipid profiles or complex metabolic conditions. Consequently, advanced lipoprotein characterization has emerged as a promising tool to enhance cardiovascular risk stratification.

Currently, a basic lipid profile must include not only the conventional measurements but also non-HDL cholesterol (determined by TC—HDLc) and apolipoprotein B determinations [12]. Despite what was previously considered, LDLc levels are not the primary determinant for developing atherosclerotic vascular disease. In recent years, growing evidence has suggested that the retention of cholesterol-rich ApoB–containing lipoproteins within the arterial wall is closely associated with the incidence of vascular events [13]. This relationship appears to be strongly influenced by both the number and size of these atherogenic particles [13]. Several studies have demonstrated the utility of ApoB measurement as a surrogate for the number of atherogenic lipoprotein particles, since ApoB is present on all of them except HDLc. In contrast, non-HDL cholesterol provides an approximate estimate of the total cholesterol content within ApoB-containing lipoproteins. Both parameters offer complementary information and are strongly correlated [14]. Furthermore, a once-in-a-lifetime Lp(a) determination should be considered to help stratify patients’ vascular risk. This is especially relevant in subjects with elevated Lp(a), since ApoB underestimates their vascular risk due to the increased atherogenicity of Lp(a) compared to that of LDLc [15].

In recent years, there has been an unstoppable development in technology that has enabled us to go beyond blood lipid tests. Mass spectrometry has revolutionized the field of lipidomics by enabling the identification and quantification of thousands of lipid species based on charge, size, mass, and shape. The use of lipidomics can, hence, help identify new lipid biomarkers associated with CVD, potentially leading to early diagnosis. This technology can also be used to find new drug targets and map out lipid metabolic pathways involved in atherogenesis [16]. Simultaneously, techniques such as nuclear magnetic resonance (NMR) spectroscopy allow for the quantification of lipoprotein particle number and size, derived from the biological sample (serum or plasma) proton NMR spectrum of the patient. Each class of lipoproteins—LDLc, HDLc, and very low-density lipoprotein cholesterol (VLDLc)—emits a unique spectral line shape that allows them to be distinguished from one another. Regarding particle quantification, NMR signal amplitude is directly proportional to the number of particles emitting the signal [17]. As we highlighted before, particle size and number may be stronger predictors of vascular risk than traditional lipid measures. Therefore, incorporating these advanced techniques into risk stratification is essential to identify individuals with elevated residual risk who may benefit from more intensive therapeutic interventions.

## 4. Novel Devices and Telemedicine in Lipidology

The development of diagnostic and monitoring technologies has significantly transformed the way cardiovascular and metabolic diseases are detected, managed, and prevented. In particular, the emergence of portable, digital, and connected tools has enabled the decentralization of risk factor management by facilitating their monitoring outside the hospital setting and promoting greater patient autonomy. In this context, innovative devices have emerged that can measure biochemical parameters, record physiological data, and support clinical decision-making through integrated systems.

Among the most relevant current strategies are electrochemical sensors capable of quantifying cholesterol levels from a small blood sample (Table 2) [18]. These are generally classified into two main categories based on their detection principle:

- Enzymatic sensors: the most established technology, employing enzymes such as cholesterol oxidase (ChOx) and cholesterol esterase (ChE). These devices, found in commercial products like CardioCheck^®^ Plus (PTS Diagnostics, Indianapolis, IN, USA) and Accutrend^®^ Plus (Roche Diagnostics, Mannheim, Germany), enable rapid measurement of total cholesterol, HDLc, LDLc, and triglycerides from a drop of capillary blood. Although they offer high sensitivity and specificity, their performance can be affected by the instability of enzymes under environmental conditions (pH, temperature). Another limitation is their high cost.
- Non-enzymatic sensors: currently under development, these systems rely on the direct oxidation of cholesterol on electrodes modified with nanomaterials such as graphene, metal oxides, or carbon nanotubes, offering greater robustness, stability, and lower potential cost.

**Table 2:** Overview of emerging and established technologies for lipid detection and monitoring.

Technology	Detection Principle	Sample Type	Advantages	Disadvantages
**Enzymatic electrochemical sensors**	Enzymatic reaction + electrochemical signal	Capillary blood	High accuracy and specificity Commercially available	Use of unstable enzymes Requires finger-prick sampling
**Non-enzymatic electrochemical sensors**	Direct oxidation on nanomaterial-modified electrodes	Blood, serum	No enzymes required (greater stability) Potentially low cost	Not yet clinically validated Require advanced materials
**Optical sensors (colorimetric, fluorescent)**	Color or fluorescence change detected via smartphone	Capillary blood, serum	Portable and low-cost Smartphone-compatible	Not validated Sensitive to light/position Require specialized reagents
**Chemiluminescent sensors**	Light emission from chemical reaction (captured by camera)	Blood, serum	High luminescent sensitivity Accurate digital readout	In preclinical studies Require accessories (dark box) Limited to controlled environments
**Microfluidic devices/lab-on-a-chip**	Electrochemical or luminescent reaction in microchannels	Blood, serum, and biological fluids	Multi-analyte from a small volume Rapid results	High manufacturing complexity Not yet commercialized
**Smart contact lenses**	Optical detection of cholesterol in tears	Tears	Non-invasive (tear-based) Highly innovative	Still experimental Not clinically available

Both types of sensors have been integrated into miniaturized lab-on-a-chip (LoC) platforms, which can operate with minimal sample volumes (microliters) and are suited for portable and multifunctional diagnostic settings. These devices incorporate microchannels for handling biological fluids and sensors that generate signals proportional to the concentration of the target analyte [18,19]. In addition, non-invasive devices such as skin patches and smart contact lenses with integrated optical biosensors have been developed to detect cholesterol in sweat and tears, respectively, with promising results in preclinical studies [20].

In parallel, mobile biosensors integrated with smartphones are being evaluated. These use colorimetric or chemiluminescent systems for cholesterol detection. In the case of colorimetric sensors, paper strips functionalized with enzymes react with the sample, producing a color change that can be quantified using the phone’s camera. Chemiluminescent sensors, on the other hand, involve chemical reactions that emit light upon interaction with cholesterol in biological samples; this light is also detected by the smartphone camera, usually under controlled conditions (using dark boxes and optical filters). Although still in the experimental phase, these technologies represent an accessible, cost-effective, and adaptable approach to self-monitoring and home-based lipid assessment, especially in low-resource settings [21].

In recent years, the growing use of wearable devices (such as smartwatches) and mobile health (mHealth) apps has opened new avenues for monitoring cardiometabolic risk. While these tools do not measure lipid levels directly, they allow for tracking of relevant physiological parameters such as heart rate, blood pressure, sleep, and physical activity. When integrated into digital platforms, these data contribute to overall risk estimation and improve adherence to lifestyle interventions [22]. In fact, some studies have shown significant reductions in LDL cholesterol using health apps combined with nutritional strategies and structured physical activity programs [23].

The added value of these technologies lies in their ability to integrate with digital health programs and telemedicine platforms, enabling continuous, personalized, and proactive monitoring of patients with dyslipidemia or at risk of developing it.

Telemedicine has gained increasing relevance in this area by facilitating continuity of care without requiring in-person visits. Digital health platforms allow healthcare professionals to access clinical data, lab results, and records from portable devices, optimizing real-time decision-making. This model has proven particularly useful during events such as the COVID-19 pandemic and in rural areas with limited healthcare access. Several recent studies have demonstrated the effectiveness of remote care programs in managing cardiovascular risk factors. For example, Blood et al. implemented a remote management program for over 10,000 patients, combining automated algorithms, laboratory monitoring, and virtual consultations to control hypertension and dyslipidemia, achieving significant clinical improvements in diverse populations [24]. Likewise, Choi et al. reported improvements in lipid profiles and postprandial glycemic control in patients with type-2 diabetes through telemonitoring strategies [25].

**Figure 2:**
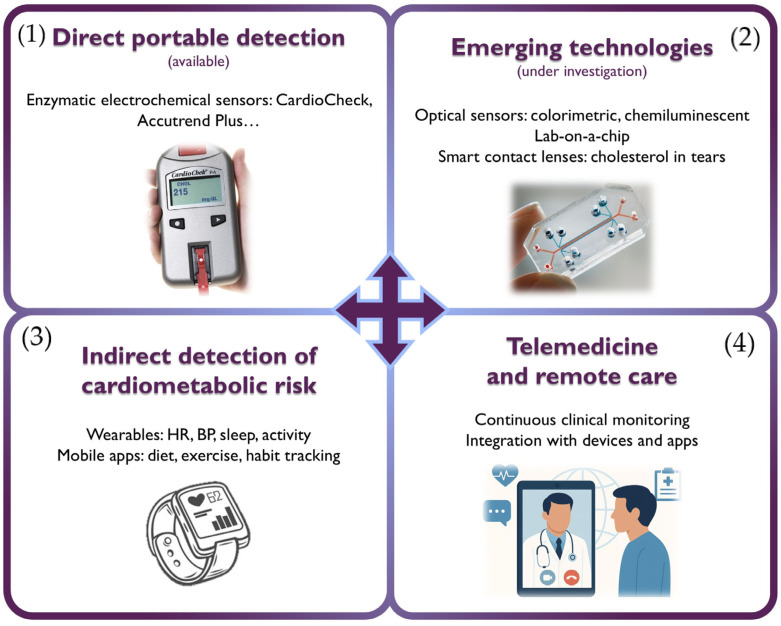
Current and emerging technologies for lipid monitoring and cardiometabolic care. The figure summarizes four complementary strategies for the diagnosis and follow-up of dyslipidemia. (**1**) Direct portable detection using enzymatic electrochemical biosensors. (**2**) Emerging technologies include optical biosensors, lab-on-a-chip platforms, and smart contact lenses for non-invasive lipid analysis in alternative fluids. (**3**) Indirect detection of cardiometabolic risk through wearable devices and mobile health apps (**4**) Telemedicine and remote care facilitate continuous clinical monitoring, integrating data from digital tools to enable proactive, personalized management of dyslipidemia. BP: Blood pressure; HR: Heart rate.

The integration of data from personal devices, mobile apps, and periodic biochemical testing into telemedicine platforms provides a comprehensive view of cardiometabolic risk, enhancing therapeutic adherence, clinical control, and healthcare system efficiency (Figure 2). In this regard, remote care is emerging as a strategic pillar in the long-term follow-up of patients with dyslipidemia and the implementation of evidence-based preventive interventions.

## 5. Emerging Lipid-Lowering Therapies

Oral lipid-lowering therapies include statins, ezetimibe, and, more recently, bempedoic acid. Despite all of them having demonstrated in clinical trials to significantly reduce the risk of cardiovascular outcomes in patients with dyslipidemia, the fact is that LDLc control rates remain far from optimal [26,27,28]. This is due to an underuse of lipid-lowering therapies, including PCSK9 inhibitors, but also because new drugs with complementary mechanisms of action are warranted [29,30].

Emerging pharmacological therapies are transforming lipid management, enabling a more targeted and precise approach. Currently, agents such as PCSK9 inhibitors (monoclonal antibodies) and inclisiran (a small interfering ribonucleic acid [siRNA]) are routinely integrated into clinical practice within numerous healthcare systems, offering potent LDLc reduction with convenient dosing schemes. Beyond these already established options, more innovations, including gene-editing technologies (CRISPR-Cas9) and nanoparticle-driven drug delivery systems, are under extensive investigation [30]. Such innovations may offer effective therapeutic alternatives for patients with dyslipidemia, particularly for conditions that are refractory to conventional therapies. However, access to emerging lipid-lowering therapies is hindered by geographical, regulatory, and affordability barriers. Policy reforms, innovative funding, education, and collaborative efforts are essential to reduce disparities and improve global access [31].

### 5.1. RNA-Based Therapies

Recent advances in RNA-targeted therapeutics have revolutionized dyslipidemia management. Inclisiran, a small interfering RNA therapy, inhibits PCSK9 synthesis via RNA interference, achieving sustained LDLc reduction with biannual dosing. It demonstrated significant LDLc reduction in phase-3 clinical trials in patients with atherosclerotic CVD or patients at equivalent risk [32].

Similarly, pelacarsen is an antisense oligonucleotide that reduces Lp(a) levels by up to 80% in phase-2 clinical trials [33,34]. Pelacarsen is complementary to the mRNA encoding apolipoprotein A (ApoA), a key component of Lp(a) synthesized in the liver. It binds specifically to the LPA mRNA, recruiting an endogenous enzyme that cleaves the target mRNA strand (RNase H1) and prevents the translation of ApoA, reducing hepatic production of Lp(a) particles [35]. Phase-3 Lp(a)-HORIZON trial (Novartis) is still ongoing, and results are expected for the first half of 2026.

Olpasiran and zerlarisan are also siRNA that have shown effective Lp(a) reduction in phase-2 clinical trials [36,37]. Although further trials are needed to fully understand their impact on CVD, these drugs have already shown promise as effective therapeutic options.

The main advantage of RNA-based therapies is that they are highly effective and offer durable effects with prolonged intervals between doses (usually 3 to 6 months), addressing adherence challenges in chronic lipid management [38].

### 5.2. Gene Therapy and CRISPR-Cas9

Gene-editing technologies like CRISPR-Cas9 may revolutionize the management of monogenic dyslipidemias. ANGPTL3 is an inhibitor of lipoprotein lipase, and loss-of-function mutations in that protein could reduce cholesterol and triglyceride levels and therefore the risk of coronary heart disease [39]. A phase-2 clinical trial has already demonstrated that zodasiran, an RNAi that blocks ANGPTL3, significantly decreases triglyceride levels at a 2-year follow-up [40].

CRISPR-Cas9-based therapies can also target ANGPTL3 [41]. In fact, in the last decade, several delivery platforms have been developed to carry CRISPR-Cas9–based editing systems of ANGPTL3 to the liver in vivo. They are based on lipid nanoparticles and allow the knockout of certain genes in order to drastically reduce cholesterol and triglyceride levels [39,42].

The VT-1001 trial (NCT05398029) is a phase-1b clinical trial assessing the safety of a CRISPR-based therapy using base editing to disrupt the PCSK9 gene in the liver, aiming to reduce PCSK9 and LDLc in patients with heterozygous familial hypercholesterolemia and uncontrolled coronary heart disease [43]. By permanently inactivating PCSK9, VERVE-101 leverages CRISPR’s precision to mimic natural loss-of-function mutations, offering a potential one-time treatment for lifelong LDLc management. The interim results were presented in 2023 at the American Heart Association Conference, achieving 39–55% LDLc reductions and 47–84% PCSK9 reductions, sustained up to 180 days [44].

### 5.3. Nanotechnology for Targeted Drug Delivery

Nanoparticles could play a transformative role in atherosclerosis treatment. They can deliver therapeutics (siRNA, anti-inflammatory drugs, statins) directly to atherosclerotic plaques via ligands, enhancing drug bioavailability at specific tissues. This way, they effectively modulate lipid metabolism, suppress inflammation, and promote cholesterol efflux [45].

LDL-mimicking nanoparticles can deliver statins directly to atherosclerotic plaques, reducing systemic side effects and preventing systemic clearance [46]. Similarly, siRNA-loaded exosomes show hepatic tropism, improving delivery efficiency, increasing LDL receptors expression, and reducing LDLc levels [47].

## 6. Integrating Artificial Intelligence and Lipidology

Medical care has evolved from a paternalistic model to an independent, patient-centered system. As science and medical knowledge have advanced dramatically in recent years, multidisciplinary medical teams, shared decision-making, and clinical scoring systems have become cornerstones of daily practices [48].

The incorporation of AI, particularly through machine learning (ML) and deep learning (DL), is poised to enable more accurate, rapid, and large-scale data analysis, introducing new tools and algorithms to support clinical decisions [49,50]. In just a few years, its methods and applications have undergone an important paradigm shift: moving away from supervised analyses of carefully curated, tabular feature sets toward a deep learning–powered, multimodal paradigm underpinned by foundation models [51]. Most recent and advanced AI systems can process raw, unstructured biometric signals and medical images and automatically discover complex representations that outperform what human-engineered features can capture.

AI is becoming an integral part of clinical workflows across a wide range of cardiovascular diseases: ischemic heart disease, heart failure, cardio-oncology, cardiac imaging, and electrophysiology [52,53,54,55,56]. As a result, ambulatory monitoring and predictive software for arrhythmia or advanced cardiac imaging are expanding rapidly. Additionally, AI and ML have been incorporated in various phases of clinical laboratory medicine [57].

Clinical lipidology, due to the close link between lipid metabolism and CVD, is well-suited for AI applications. AI algorithms can improve diagnostic accuracy, refine risk stratification, assess care quality, and predict outcomes. They also support precision medicine by enabling personalized treatment plans to achieve target lipid levels and by identifying new biomarkers for primary and secondary prevention of cardiovascular events.

With growing knowledge of metabolic and molecular pathways and expanding treatment options beyond statins, a wide range of AI-based tools can be envisioned (Table 3):

Some clinical applications of AI are discussed in the following sections.

### 6.1. Early Diagnosis of Familial Hypercholesterolemia

Familial hypercholesterolemia (FH) is the most prevalent inherited lipid disorder, affecting about 0.32% of the population (1 in 313) [58]. If left untreated, FH increases coronary artery disease risk by 10–17-fold versus non-FH individuals and leads to earlier cardiovascular events due to persistently high LDL-cholesterol levels [59]. Diagnosis follows the Dutch Lipid Clinic Network (DLCN) criteria, which combine clinical signs and laboratory results [60], and can be confirmed by identifying pathogenic variants in LDLR, APOB, or PCSK9 [61,62]. Other clinical criteria that have been validated for FH diagnosis are the Make Early Diagnosis to Prevent Early Deaths (MEDPED) [63], American Heart Association [64], and Simon Broome [65].

FH remains highly underdiagnosed [66]. Machine learning algorithms that automatically screen electronic health and laboratory records offer a promising solution (Table 4) [67]. The Family Heart Foundation’s FIND FH model was one of the first validated algorithms trained on electronic health records (EHR) data from clinically diagnosed FH patients [68]. It applied a supervised learning approach to individuals with at least one CVD or primary prevention comorbidity claim, using structured clinical data. The model was intended to differentiate those with clinically diagnosed FH from those presumed without FH. Training involved sequential random-forest layers to select the top-ranked features and then computing an FH score. The performance metrics were as follows: precision (positive predictive value) 0.85, recall (sensitivity) 0.45, area under the precision–recall curve (AUPRC) 0.55, and area under the receiver operating characteristic curve (AUROC) 0.89. An expert reviewed sample of individuals flagged by the algorithm in two external validation cohorts confirmed that 77% and 87% of them presented high enough clinical suspicion of FH.

A natural language processing (NLP) based algorithm for EHR data mining was developed by the Mayo Clinic in 2016 and externally validated alongside the FIND FH model by Gidding et al. [69] using the Geisinger MyCode Community Health Initiative cohort (n = 130,257), both algorithms achieved a 70% net genetic diagnosis yield for FH, though phenotypic diagnosis was limited by missing physical-exam and family history data. Kim et al. [70] further tested the FIND FH model in a large U.S. health system. 32 out of 471 patients flagged by the algorithm met formal phenotypic criteria on manual review, and one-third were judged highly likely to have FH but were not receiving intensive lipid-lowering therapy. Banda et al. [71] also developed a screening algorithm as part of the FIND FH initiative using a random forest classifier trained on EHR data, obtaining an outstanding AUROC of 0.94% in an external validation cohort.

Akyea et al. [72] assessed the performance of five common machine-learning algorithms (logistic regression, random forest, gradient boosting machines, neural networks, and ensemble learning) for detecting FH within a large primary care population. Four of these methods demonstrated high predictive accuracy and helped identify additional features apart from known clinical features of FH. However, although the negative predictive value of all algorithms was above 99%, the highest positive predictive value was only 15.5%. Another example of classic machine learning methodology is the study by Gratton et al. [73], where they identified a combination of 14 features, including an LDL polygenic score, by using a Least Absolute Shrinkage and Selection Operator (LASSO) technique with a predictive performance by AUC of 0.77.

These algorithms seem to offer better performance than clinical scores. Pina et al. [74] published their results using three different ML algorithms in two independent FH cohorts. External validation showed greater AUROC for classification trees, gradient-boosting machine, and neural networks than the DLCN. A combination of logistic regression, deep learning, and random forest using age, sex, and lipid profile results demonstrated better performance (AUROC 0.71) than a simple LDLc cutoff (AUROC 0.64) and comparable to the DLNC criteria (AUROC 0.70) in a validation cohort of genetically confirmed FN [75].

All of these studies concur in the difficulty of adequately classifying these subjects as “definite” FH patients, due to the lack of a formal definition and the fact that many patients do not present with pathogenic variants of the described genes. Khademi et al. [76] published a preprint version of their manuscript where they adopt a multi-class approach to FH detection, considering four categories (Definite, Probable, Possible, and Unlikely). They proposed a TabNet architecture that takes advantage of the most recent ML algorithms, such as tabular Deep Neural Networks and feature and attentive transformers. After five-fold cross-validation, the model demonstrated excellent performance with an AUC ranging between 0.84 and 0.95. However, it was still trained on tabular, structured data and has not yet undergone peer review or external validation.

Another common limitation related to the uncertain diagnostic criteria is that a proper external validation still requires laborious chart review. Hence, prospective implementation studies that run these tools in parallel with routine care are warranted [67].

**Table 4:** AI-enhanced identification of familial hypercholesterolemia.

Author (Year)	Cohort (s)	Algorithm (s)	Results
**Myers et al. (2019)** [68]	Training: EHR data from 939 clinically diagnosed individuals with FH and 83,136 controls sampled from four U.S. institutions Validation: National healthcare encounter database (170 million individuals) and an integrated healthcare delivery system dataset (174,000 individuals)	FIND HF (sequential random forest)	Internal validation: Precision 0.85, Recall 0.45, AUPRC 0.55, AUROC 0.89 External validation: 87% of the flagged individuals in the national database and 77% in the healthcare delivery system dataset were categorized as having high clinical suspicion of FH.
**Banda et al. (2019)** [71]	Training: 3.1 million patients (197 known FH patients and 6590 selected controls) Validation: Geisinger Healthcare System dataset (33,086 patients, 466 cases)	Random forest classifier	Internal validation: Positive predictive value (PPV) of 0.88 and sensitivity of 0.75 External validation: PPV of 0.85
**Akyea et al. (2020)** [72]	Training: 3,020,832 individuals from the Clinical Practice Research Datalink (United Kingdom) Validation: 1,006,943 individuals from the same dataset	Logistic regression Random forest Gradient boosting Deep learning Ensemble model (combination)	External validation: Every model had AUC > 0.89 except for logistic regression (AUC 0.81). Ensemble learning demonstrated a high likelihood ratio (45.5)
**Pina et al. (2020)** [74]	Training: FH Gothenburg cohort (174 patients) Validation: FH-CEGP Milan cohort (364 patients)	Classification tree Gradient boosting Neural network	Internal validation: AUROCs: 0.79 (CT), 0.83 (GBM), 0.83 (NN), 0.68 (DLCN) External validation: AUROCs: 0.70 (CT), 0.78 (GBM), 0.76 (NN), 0.64 (DLCN)
**Hesse et al. (2022)** [75]	Training: Charlotte Maxeke Johannesburg Academic Hospital’s (678 patients) Validation: Groote Schuur Hospital (1376 with clinical FH and 2655 with potential FH)	Logistic regression Deep learning Random forest	External validation: AUROC 0.711 in a high-prevalence FH sample (DLCN 0.705) and 0.801 in a medium-prevalence FH sample
**Gratton et al. (2023)** [73]	Training: UK Biobank (139,779 individuals)	Least absolute shrinkage and selection operator logistic regression	Internal validation: 14 variable models with AUC of 0.77
**Gidding et al. (2023)** [69]	Validation: Geisinger MyCode Community Health Initiative cohort (59,729 individuals)	FIND FH (random forest) Mayo Clinic (NLP)	External validation: 5.9% of 573 flagged by FIND FH and 1.9% of 10,415 by Mayo had a pathogenic or likely pathogenic (P/LP) variant. At least one algorithm identified 197 out of 280 individuals with P/LP variant (net yield: 70%)
**Kim et al. (2024)** [70]	Validation: 167,955 individuals from the Oregon Health and Science University Health Care System	FIND HF (random forest)	External validation: 121/471 flagged individuals met “likely” FH criteria and received suboptimal lipid-lowering therapies
**Khademi et al. (2024)** [76]	Training: 1591 individuals	Multi-Stage Tabular Deep Learning Network (FH-TabNet)	Internal validation: F1-score 98.2, 98.6, 87.2, and 19.2 for “Unlikely”, “Possible”, “Probable”, and “Definite” FH class prediction

AUPRC: Area under the precision-recall curve; AUROC: Area under the receiver operating characteristics curve; DLCN: Dutch lipid clinic network score; EHR: Electronic health record; FH: Familial hypercholesterolemia; NLP: Natural language processing.

### 6.2. Enhanced LDLc Estimation

Machine learning approaches have been developed in an effort to improve the performance of traditional formulas for LDLc estimation, such as the Friedewald [77], Martin–Hopkins [78], or Sampson [79] equations (Table 5).

Singh et al. [80] employed random forests to train a model that demonstrated slightly improved performance (R^2^ = 0.982) when estimating directly measured LDLc, outperforming both the Friedewald and Martin–Hopkins equations (R^2^ = 0.950 and 0.962, respectively).

The performance of other commonly used machine learning algorithms, such as K-nearest neighbors (KNN), has also been assessed in two independent studies. Barakett-Hamade et al. [81] found that their LDL-KNN model provided better accuracy than the Friedewald and de Cordova formulas, regardless of triglyceride levels. However, it underperformed when compared to the Martin–Hopkins and Sampson equations. Ghayad et al. [82] subsequently validated this model in a dataset comprising 38,452 observations and reported intraclass correlation coefficients above 0.9 in most cases. Nonetheless, performance was notably limited in samples with triglyceride levels >400 mg/dL or markedly reduced LDLc concentrations (<40–55 mg/dL). Unfortunately, no direct comparison with classical formulas was included in their analysis.

Cubuku et al. [83] addressed these limitations by demonstrating improved estimation in extreme cases (e.g., very high triglycerides or low LDLc) using linear regression, gradient-boosted trees, and deep neural networks (DNNs), all of which outperformed traditional formulas. These findings were replicated by Oh et al. [84], who built additional gradient boosting and neural network models.

Deep learning approaches have also been explored by Lee et al. and Tsigalou et al. [85], who both used DNNs to estimate LDLc. They confirmed superior accuracy compared to conventional equations but also cautioned about the computational cost of DNNs, suggesting that simpler machine learning algorithms—such as linear regression, support vector machines, or extreme gradient boosting—may offer similar performance with greater efficiency [85].

In another study, Kwon et al. [86] developed a DNN specifically for the Korean population, achieving higher concordance with directly measured LDLc than the Friedewald, Martin, or Sampson formulas. However, inaccuracies persisted in cases with elevated triglyceride levels and LDLc values below 100 mg/dL.

Finally, the performance of machine learning models appears to be highly dependent on the training dataset, as shown by Hidekazu et al. [87] Their comparison of nine different ML algorithms across three distinct datasets revealed that models trained on clinical patient data showed greater deviations from reference LDLc values than those trained on health check-up populations. A generalized adoption of these new estimation tools will require population-specific external validation and practical ease of implementation, balancing precision with computational efficiency and thus enhancing cardiovascular risk prediction.

**Table 5:** AI-driven solutions for LDLc estimation.

Author (Year)	Cohort (s)	Algorithm (s)	Results
**Singh et al. (2020)** [80]	Training: 17,500 lipid profiles performed on 10,936 individuals (New York-Presbyterian Hospital/Weill Cornell Medicine)	Random forests (Weill Cornell model)	Internal validation: Correlation coefficients between estimated and measured LDLc were 0.982 (Weill Cornell model), 0.950 (Friedewald), and 0.962 (Martin–Hopkins).
**Barakett-Hamade et al. (2021)** [81]	Training: 31,922 observations from 19,279 subjects	K-nearest neighbors (LDL-KNN)	Internal validation: Intraclass correlation coefficients: 0.937 (Martin–Hopkins), 0.935 (Sampson), 0.925 (LDL-KNN), 0.894 (Friedewald), 0.869 (de Cordova),
**Tsigalou et al. (2021)** [85]	Training: 4244 records (Sismanoglio General Hospital of Komotini) Validation: 478 records (Democritus Diagnostic Center in Alexandroupolis, University Hospital of Alexandroupolis)	Linear regression (LR), Support Vector Machines (SVM), Extreme Gradient Boosting (XGB), Deep Neural Networks (DNN)	External validation. Simple ML techniques can work as well as neural networks. Standard error of the estimate (the lower, the better): - Cohort 1: 13.3 (LR), 10.1 (SVM), 15.6 (XGB), 10.1 (DNN) - Cohort 2: 20.9 (LR), 33.4 (SVM), 21 (XGB), 34.4 (DNN)
**Ghayad et al. (2022)** [82]	Training: 31,853 retrospective records (Hotel Dieu de France University Hospital) Validation: 6599 prospective records (Hotel Dieu de France University Hospital)	LDL-KNN	External validation: ICCs above the 0.9 cutoff except for low LDLc or very high triglycerides
**Çubukçu et al. (2022)** [83]	Training: 59,415 records (Başkent University)	Linear regression (LR), gradient-boosted trees, artificial neural network (ANN)	Internal validation. Better performance than classical formulas for LDLc < 70 mg/dL and TG > 177 mg/dL [F1-scores: 70.46 (ANN), 69.6 (gradient-boosted trees), 69.23 (LR), 62.71 (Martin–Hopkins), 62.38 (Friedewald)]
**Oh et al. (2022)** [84]	Training: 823,657 records (Seoul National University Hospital)	Gradient boosting (LDL-C^X^), neural network (LDL-C^N^)	Internal validation. Better correlation compared to traditional formulas [overall bias: −0.27 mg/dL (LDL-C^X^), −0.01 mg/dL (LDL-C^N^), −3.80 mg/dL (Friedewald), −2.00 mg/dL (Martin–Hopkins)]
**Kwon et al. (2022)** [86]	Training: 129,930 records (Gangnam Severance Health Check-up) Validation: 46,470 records (Korean Initiatives on Coronary Artery Calcification registry)	Deep neural network (DNN)	External validation: The DNN method had lower bias and root mean-square error than Friedewald’s, Martin’s, and Sampson equations

ICC: Intraclass correlation coefficient; ML: Machine learning; LDLc: Low-density lipoprotein cholesterol.

### 6.3. Statin Use and Target LDLc Achievements

Achievement of LDLc thresholds among patients at high or very high cardiovascular risk remains suboptimal, primarily due to therapeutic inertia and underutilization of high-intensity statins and adjunctive lipid-lowering therapies [88]. The prevalence of statin intolerance is likely overestimated when applying broad international definitions and should be considered a marginal barrier to statin initiation [89].

Masi et al. [90] conducted an analysis of lipid profiles from over eleven thousand patients using a Logic Learning Machine to identify variables predictive of achieving an LDLc target below 100 mg/dL within two years. Nearly 40% of patients failed to meet this treatment goal, underscoring the persistent challenges in lipid management. The model identified LDLc levels at treatment initiation and at six months as the strongest predictors of success. Additional important variables included glycated hemoglobin, fasting glucose, body mass index, HDLc, albuminuria, male sex, younger age, use of antihypertensive medication, number of follow-up visits, and absence of therapy discontinuation. However, the study did not include a comparison with traditional logistic regression models.

Han et al. [91] developed machine learning models (XGBoost, random forest, logistic regression) using electronic health records of patients with coronary artery disease treated with moderate-intensity statins. These models achieved AUC values of approximately 0.70. Notably, the use of model explainability techniques (SHAP values) highlighted key predictors —such as baseline lipid levels and specific medication use—enabling a better understanding of individual predictions. This approach not only estimated the likelihood of LDLc target attainment but also helped identify patients who might benefit from escalation of lipid-lowering therapy.

A key determinant of poor LDLc control is non-adherence to statin therapy. Sarraju et al. [92] investigated the underlying reasons for statin discontinuation by applying a deep learning natural language processing (NLP) algorithm to EHR data. Their model achieved an AUC of 0.99 for detecting statin nonuse and 0.90 for classifying the reasons into patient-level factors (side effects, contraindications, and statin hesitancy), clinician-level factors (guideline-discordant prescribing), and system-level factors (clinical inertia).

## 7. Digital Models and Clinical Decision Support Systems

Lipid disorders significantly increase the risk of CVD, which is the main cause of mortality worldwide. Their effective management is critical to improving health outcomes. Therefore, new digital technologies applied to lipid management, particularly Clinical Decision Support Systems (CDSSs), are revolutionizing medical practice by integrating clinical and lipid data to guide therapeutic decisions more accurately. These CDSS analyze data and provide recommendations, alerts, and other support based on scientific evidence and clinical experience. They are, therefore, novel tools that help healthcare professionals deliver the best possible care to patients, improving safety and quality, as well as offering greater efficiency [93]. CDSS can operate independently (stand-alone systems) or be connected to an electronic health record database (integrated EHR systems).

They assist in several areas such as early disease detection, diagnosis, data interpretation, treatment planning, patient care, monitoring, and prognosis [94]. In this way, they are able to act in estimating treatment efficacy or risk level, recommending treatment or referral, predicting drug interactions and dosing alerts, establishing follow-up, and shared decision-making. Among the functions included are digital alerts, pop-up windows, and notifications, active or passive [95].

Since its beginning in the 1970s, multiple systems with different modalities have been developed and deployed. In addition, the use of AI for decision support systems in CVD is rapidly evolving, providing more accurate and personalized information to healthcare professionals [96].

CDSS have been associated with a positive impact on the quality of clinical practice and patient adherence in chronic disease management [94,97,98]. A meta-analysis concludes that it increases the proportion of patients who received the desired care and significantly increases the proportion of patients who achieved guideline-based goals (blood pressure or lipid control) [99].

Focusing on lipid management, recent evidence highlights several relevant initiatives in this field. The HTE-DLP is a pilot study to assess this CDSS system to improve the treatment of hyperlipidemia, providing specific therapeutic recommendations. Positive results were obtained; 55% of patients in the intervention group achieved LDLc levels <70 mg/dL, compared to 12.5% in the control group, resulting in a significant improvement in lipid control [100]. On the other hand, the MedicBK CDSS analyses information from EHR to assess cardiovascular risk and suggest treatments based on clinical guidelines and randomized clinical trials, facilitating adherence to current guidelines and optimizing the use of clinical data [101]. In addition, CDS-FH is a CDSS targeted for familial hypercholesterolemia. Integrated into EHRs, it alerts physicians to patients at elevated risk, suggesting referrals to specialists and adjustments in lipid-lowering therapy [102]. In addition, in the pediatric population, it has been shown that the implementation of electronic alerts in EHRs can increase orders and performance of lipid screening tests, improving adherence to clinical guidelines [103].

In addition, data visualization tools, such as dashboards, promise an important role. An exploratory review highlighted that combining education, collaborative modelling, and decision support systems improved clinical experience and population-based lipid management. However, the direct impact on clinical outcomes and cost reduction remains to be demonstrated [104].

Regarding the overall effectiveness of CDSS in cardiovascular prevention, one systematic review and meta-analysis found that these systems have a positive effect on blood pressure control and improved statin prescription, but no significant impact on lipid or glycemic levels [105]. Another showed beneficial trends for CDSS, although it only reached statistical significance for the achievement of LDLc targets in patients with diabetes [106]. Heterogeneity in CDSS designs limits the feasibility of drawing universal conclusions.

On the other hand, passive and active EHR intervention alerts to specialized consultations had limited effectiveness in influencing statin prescribing. A positive effect was only observed in the subgroup of patients with clinical atherosclerotic CVD [107].

Despite the potential of CDSS to improve patient care delivery and cardiovascular health outcomes, several barriers hinder their successful implementation, thus limiting their effectiveness in achieving health goals. The main barriers are time and resource limitations, alertness fatigue, technical difficulties with the CDSS system, lack of confidence in its recommendations, and the complexity of real-world clinical management of CVD. The presence of these barriers may contribute to the heterogeneity of results across studies.

In terms of the applicability of these systems, we must bear in mind that the effectiveness of the evidence is context-dependent and that outcomes are largely specific to healthcare settings and patient populations. CDSSs have the potential to improve shared decision-making, treatment adherence, and ultimately health outcomes. They are therefore a potential tool to improve the quality of our care outcomes in CVD, and specifically in lipid management. Further research in this area is needed.

## 8. Ethical, Regulatory, and Implementation Challenges

As previously discussed, the emergence of AI in lipid assessment has the potential to significantly enhance daily clinical practice, ultimately benefiting patients. The integration of AI-assisted decision-making should follow a structured approach. First, it must be conceptualized within a clear clinical framework; second, the physician must fully understand the model, including its purpose, validation process, structure, type, and potential biases; and third, the model must be continuously updated, calibrated, and modified based on new data inputs [108].

As a result, large-scale implementation of AI algorithms is difficult owing to many barriers, including the interpretability and the generalizability of the AI-based model, the adoption of new technologies by sanitary professionals, as well as technical and regulatory/ethical issues. In fact, although the AI-based algorithms have been tested in clinical studies, real implementation is challenging [109]. Thus, while some tools are simple to implement and raise minimal concerns (alarms when a patient is outside of therapeutic targets), others must be validated by a physician and pose specific challenges that must be addressed [110].

### 8.1. Information and Biases

The widespread adoption of AI will introduce concepts rooted in data science and statistics. Thus, physicians must make a considerable effort to understand the associated methodologies and terminology to prevent misinterpretation and bias [49]. A large volume of related literature is expected, necessitating a critical appraisal of publications, especially those addressing unfamiliar technical aspects. Bias may originate at various stages: in datasets used during training (data bias), as the quality of the model depends directly on the data input quality; in the development of the model or algorithms (development bias); or through clinical interpretation (interaction bias). Awareness of common sources of bias and a multidisciplinary approach are essential to minimize these risks [111,112,113]. Developing AI models should not be an end in itself; instead, efforts must focus on real-world applications with clearly defined, patient-centered objectives, avoiding “AI-hype” and commercial-driven initiatives [114].

### 8.2. Resources

Implementing AI technology in healthcare will require a substantial investment. New professional roles—such as data managers, experts, statisticians, or programmers—will be necessary to handle data input, manage sensitive information, and develop or maintain software. Furthermore, AI requires advanced digital infrastructure and extensive data sources. There are also concerns about the ownership of these programs and how health systems will purchase or lease them. Altogether, this represents a significant financial challenge for national healthcare systems and insurance providers. Although no specific cost-effective studies have been carried out at present, it is expected that the implementation of new technologies will result in better and earlier diagnosis and treatment of patients and therefore in cost savings in the medium term. Additionally, AI systems consume considerable environmental resources, making sustainability a critical consideration as these technologies become embedded in everyday practice. New technologies are currently being developed aimed at reducing this high consumption [115].

### 8.3. Ethics

In parallel with AI development, some authors have emphasized the need to define specific ethical principles that complement the traditional four pillars of medical ethics. AI tools must prioritize beneficence, particularly in reducing morbidity and mortality. Privacy must be safeguarded, with specific informed consent processes in place when personal data are included in databases. Algorithm transparency is essential to ensure that AI-driven decisions are interpretable. Algorithms should be trained using a diverse population across sex, age, socioeconomic status, and ethnicity to avoid systemic bias. Equity and social justice must be at the forefront to ensure these technologies benefit all patients, not just select groups [116,117]. Various AI checklists have been proposed to improve data quality and utility, many based on the FAIR principles—Findability, Accessibility, Interoperability, and Reusability [118].

### 8.4. Epistemic and Ontological Considerations

Although they differ from their etymological origins, the terms epistemic and ontological AI refer to distinct concepts: the former relates to the origin, nature, and validity of knowledge, while the latter involves machine-readable, manipulable, and shareable concepts. The incorporation of AI introduces a fundamentally different care model from previous ones, posing challenges that humanity has never faced before. While the utility of AI is undeniable, neither patients nor physicians should disregard clinical judgment in favor of AI-generated recommendations, particularly when they lack the nuanced “clinical eye”. Some of the information generated by AI may lack clinical significance. On the one hand, healthcare professionals should not be constrained or misled by AI models, especially when they do not fully understand them or when the data used is inappropriate or biased—as previously discussed, these models are not free from bias. On the other hand, patients must not be reduced to digital checklists or multiple-choice questionnaires, becoming “e-patients”, as their personal and human dimensions are far beyond that. Reflective decision-making, supported by AI, between patients and healthcare providers must be preserved [119,120].

### 8.5. Validation and Legal Frameworks

Interoperability remains a major challenge for many AI systems. Tools designed for a limited number of institutions will have restricted scalability. To promote broader adoption, external and prospective validation is essential. Given the growing number of AI algorithms, conducting large-scale clinical trials is increasingly difficult. Pragmatic trials evaluating real-world effectiveness may serve as a viable alternative [114]. The attribution of responsibility in cases where errors arise from machine learning–assisted clinical decisions remains ambiguous, particularly between the clinician and the system’s developer [121]. Health systems were not initially structured to accommodate AI/ML. However, agencies such as the U.S. Food and Drug Administration (FDA) have recognized this and are actively publishing updated guidance on best practices, transparency, and AI-enabled medical devices [122]. The European Medicines Agency (EMA) also has released a “Reflection paper on the use of AI in the medical products lifecycle” and has initiated a multi-year work plan aiming for comprehensive regulatory integration by 2028 [123].

### 8.6. Patient Perspective

Although efforts of AI-based algorithms are mainly focused on improving the management of patients and evidence highlights the impact of AI on health care, data about patient perspectives of AI is scarce. However, patients are key stakeholders essential to the proper implementation of AI in health care. In fact, patients’ needs and expectations are not routinely considered in the application of AI in health care. Therefore, there is a need to include patient voices in the development of AI to ensure good implementation and application in clinical practice [124].

## 9. Conclusions

**Figure 3:**
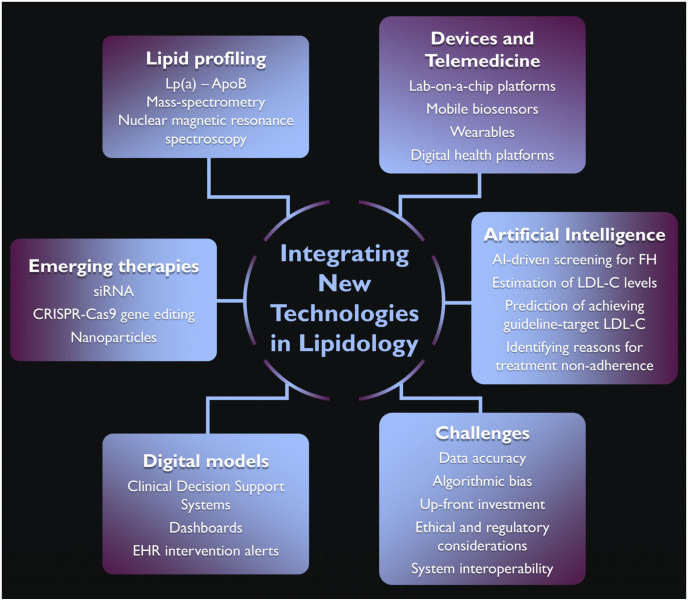
Central Illustration. Integrating Artificial Intelligence in Lipidomics.

Technological innovation is reshaping every facet of lipidology (Figure 3). Advanced lipidomics, NMR particle profiling, and cardiovascular imaging now complement the conventional lipid panel, unmasking residual risks that standard metrics fail to detect. Portable electrochemical and optical biosensors, wearables, and telemedicine platforms decentralize monitoring and enable earlier intervention and greater patient autonomy. The new therapeutic strategies will offer more durable effects and improved adherence; thus, they are expected to play a key role in reducing the global burden of cardiovascular disease. AI models are able to effectively detect familial hypercholesterolemia, refine population-level LDLc estimation, and power clinical-decision support systems. Coupled with multi-omics and imaging data, they herald a new era of precision cardiometabolic care. Robust external validation, transparent algorithms, interoperable data architectures, and agile regulatory pathways are essential to translate proof-of-concept tools into equitable, scalable solutions. In summary, integrating new technologies into lipidology intends to move the field beyond an “LDL-centric” paradigm toward a holistic, continuously learning system that can precisely quantify risk, intervene earlier, and tailor therapy across the life-course.

## Figures and Tables

**Table 1:** Global trends in cardiovascular (CV) mortality from 2025 to projected estimates for 2050. CV: Cardiovascular. Image performed with data from Chong et al. [2].

Year	CV Deaths/Year	Variation 2025–2050		
1990	12.1 million	CV prevalence	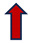	90.0%
		Age-standardized cardiovascular prevalence	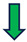	3.6%
2019	18.6 million	Total CV deaths	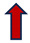	73.4%
2050	35.6 million	Age-standardized CV mortality rates	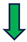	30.5%

**Table 3:** Integrating Artificial Intelligence into Clinical Lipidology.

Integrating AI into Clinical Lipidology
Automatic laboratory alerts when values fall outside guideline-based thresholds.
Integrated tools combining LDLc, total cholesterol, ApoB, non-HDL, VLDLc, remnant particles, Lp(a), and glucose-triglycerides index to suggest optimal treatment strategies.
AI-assisted drug discovery through protein structure prediction
Improved models for predicting cardiovascular events in diverse populations
Advanced statistical modelling in clinical trials to sample size and enable automatic analysis.
Enhanced prediction of familial hypercholesterolemia and deeper insight into inheritance patterns, including non-Mendelian traits.
Risk scores for primary prevention that incorporate broader clinical and laboratory data to guide early intervention or further diagnostic evaluation.
Personalized dietary and physical activity recommendations for patients.

LDLc: Low-density lipoprotein cholesterol; VLDLc: Very-low-density lipoprotein cholesterol; Lp(a): Lipoprotein (a); ApoB: Apolipoprotein B.

## Abbreviations

AI	Artificial Intelligence
ANGPTL3	Angiopoietin-Like protein 3
ApoB	Apolipoprotein B
AUC	Area Under the Curve
AUROC	Area Under the Receiver-Operating-Characteristic Curve
AUPRC	Area Under the Precision–Recall Curve
CDSS	Clinical Decision-Support System
CDS-FH	Clinical Decision-Support for Familial Hypercholesterolemia
CRISPR	Clustered Regularly Interspaced Short Palindromic Repeats
CT	Computed Tomography
CV	Cardiovascular
CVD	Cardiovascular Disease
DL	Deep Learning
DLCN	Dutch Lipid Clinic Network
EHR	Electronic Health Record
EMA	European Medicines Agency
FDA	Food and Drug Administration
FH	Familial Hypercholesterolemia
HDLc	High-Density Lipoprotein Cholesterol
KNN	k-Nearest Neighbours
LASSO	Least-Absolute-Shrinkage-and-Selection Operator
LDLc	Low-Density Lipoprotein Cholesterol
LDLR	Low-Density Lipoprotein Receptor
Lp(a)	Lipoprotein(a)
ML	Machine Learning
MRS	Magnetic-Resonance Spectroscopy
NMR	Nuclear-Magnetic Resonance
NLP	Natural-Language Processing
PCSK9	Proprotein-Convertase Subtilisin/Kexin type 9
siRNA	Small-Interfering RNA
TC	Total Cholesterol
TG	Triglycerides
